# The Federated Practice of Soccer Influences Hamstring Flexibility in Healthy Adolescents: Role of Age and Weight Status

**DOI:** 10.3390/sports8040049

**Published:** 2020-04-13

**Authors:** Jesús Gustavo Ponce-González, José V. Gutiérrez-Manzanedo, Guillermo De Castro-Maqueda, Victor Jose Fernández-Torres, Jorge R. Fernández-Santos

**Affiliations:** 1 MOVE-IT Research Group and Biomedical Research and Innovation Institute of Cádiz (INiBICA) Research Unit, Puerta del Mar University Hospital University of Cádiz, 11009 Cádiz, Spain; jesusgustavo.ponce@uca.es; 2 Department of Physical Education, Faculty of Education Sciences, University of Cádiz, 11519 Puerto Real, Spain; guillermoramon.decastro@uca.es (G.D.C.-M.); victorjfdez@yahoo.es (V.J.F.-T.); jorgedelrosario.fernandez@uca.es (J.R.F.-S.)

**Keywords:** adolescents, deep trunk flexion, sit and reach test, soccer

## Abstract

The aim of this study was to compare the hamstring flexibility between federated soccer and non-federated adolescents, and also to evaluate the effect of age and weight status on hamstring flexibility. The participants were 234 students (11–18 years old) divided into: (i) G1: non-federated (n = 127), and (ii) G2: federated in soccer (n = 107). The deep flexion of the trunk (DF) test and the sit and reach test (SRT) were performed. G2 showed higher values for the DF and SRT compared to G1 (*p* < 0.05). Both flexibility tests correlated positively (r = 0.4, *p* < 0.001). Body mass index (BMI) was negatively correlated with the DF test (r = −0.3, *p* < 0.001), but not with the SRT. Divided by BMI, the underweight and normal weight groups had higher scores in the DF test compared with the overweight and obese groups (*p* < 0.001). BMI was negatively correlated with hamstring flexibility. Federated soccer students present higher scores of hamstring flexibility.

## 1. Introduction

Flexibility is traditionally considered as the joint range of motion and has been considered as an important element of physical fitness and good health from 1980 [1] to the present [2,3]. Benefits of flexibility can be obtained with stretching exercises that are associated with improvements of range of motion and function, improved athletic performance, reduced injury risk, prevention or reduction of post exercise soreness and improved coordination [4]. Moreover, low hamstring flexibility has been associated with lower back pain in adolescents [5,6,7] and is associated with a higher risk of lower back pain later in life [8]. However, the influence of physical activity outside of school physical education programs (i.e., federated sports) on hamstring flexibility in adolescents remains unknown.

There is a decline of physical activity and an increase in sedentary behaviors from childhood to adolescence, which can increase the risk of obesity [9]. There is a controversy about the association between fatness and hamstring flexibility measured with the sit and reach test, where some authors showed no significant association [2,10,11,12] and others found significant positive association [13]. The sit and reach test is widely used to assess hamstring flexibility in health-related fitness test batteries and is also used in youths since it has a simple procedure, is easy to administer and requires minimal skill training [2]. However, whereas several studies measure the sit and reach test, less information is available regarding the deep flexion of the trunk test, which is focused on the lower back [14,15], and its relationship with fatness is unknown. 

One of the aims of the contents of the curriculum in secondary education is the improvement of basic physical qualities, such as body composition and flexibility. Hence, it is important to determine if students who only perform physical activity during curricular classes have the same weight status and hamstring flexibility compared to students who do extracurricular sports practice in a federated way. 

The most frequently played sport worldwide during childhood is soccer, with more than 270 million federated and non-federated practitioners of either sex and of all ages [16]. The regular practice of soccer has been suggested to be beneficial for many health aspects, since regular exercise can reduce the risk of many diseases. However, soccer has an elevated risk of injury, due in part to the high intensity actions and elevated forces and joint loads [17]. Good flexibility is a marker of general physical wellbeing but is particularly important for many athletes as an intrinsic risk factor for musculoskeletal injuries. Lack of flexibility in the hamstring muscle is one of the most commonly postulated risk factors for the development of musculoskeletal injuries, especially in soccer [18,19,20], with an incidence of injuries of 17% in the thigh and 8% in the groin (68%–88% in the lower extremities) [20]. However, little is known about the influence of soccer practice in a federated team on hamstring flexibility during adolescence.

The aim of this study was to compare the hamstring flexibility between federated footballers and non-federated adolescents and to investigate its relationship with age and weight status.

## 2. Material and Methods

### 2.1. Participants

In this cross-sectional study, we recruited 250 Caucasian male adolescents in random order belonging to high school (11 to 17.9 years of age). Of all participants, 16 were excluded because they did not meet the inclusion/exclusion criteria (total participants = 234 students). The exclusion criteria were having acute lumbar pain (2 participants), musculoskeletal injury in the leg (2 participants), a structured spinal injury previously diagnosed, and/or to be federated in any other sports discipline except soccer (12 participants). In addition, belonging to a federated soccer team implies that students are training regularly during the week with the supervision of a capable professional, are competing regularly in matches, and had to meet the following inclusion criteria: (i) to belong to a federated soccer team for at least two years (2 students), and (ii) to train between 3 or 4 days/week with sessions among 60–120 min. All participants were instructed not to do any physical exercise in the 24 hours prior to the measures to avoid any kind of influence.

In order to conduct the analyses for the factor invariance, the sample was divided into two sub-samples: (a) non-federated: students who were not involved in any federated sport (n = 127) and (b) federated: students belonging to a federated soccer team (n = 107).

All participants and legal tutors were informed about the protocol of the study and the experimental risks and benefits of their participation. The students gave their assent and their parents signed the written consent forms for participating in the research. The students were also informed of the right to refuse participation in the study at any time. None of the students and parents reported any discomfort in participating in this research. This study was conducted in accordance with the principles of the Declaration of Helsinki (Fortaleza, Brasil, October 2013) and the International Council on Harmonization Guidelines for Good Clinical Practice, as well as the Spanish legal framework for clinical research on humans (Real Decreto 561/1993 on clinical trial). Ethics approval has been provided by the Ethics Committee for Research Involving Human Subjects at the University of Cadiz in 2011 upon request by the principal investigator (PI) of this study, PhD. Guillermo de Castro Maqueda, who performed all the measurements.

### 2.2. Measures

The measurements were performed over 2 consecutive weeks in April 2012. Each participant had only one day of testing, which was performed during Physical Education classes at the school timetable in the same condition (see procedures). 

*Body mass index (BMI).* Height and body mass were measured while barefoot, wearing shorts and t-shirt. Height was measured to the nearest 0.1 cm using a stadiometer (Holtain, Crymmych, UK) and body mass was assessed to the nearest 0.1 kg using a Seca scale (SECA 861, Leicester, UK). Instruments were calibrated to ensure acceptable accuracy. BMI was calculated as body mass/stature squared (kg/m^2^) and categorized by the BMI international cut-off values as underweight (UW), normal weight (NW), overweight (OW), and obese (OB) according to Cole et al. [21,22].

*Hamstring flexibility.* This component was evaluated using the sit and reach test (SRT) following the protocol described by Ayala, Sainz de Baranda, De Ste Croix & Santoja [23]. It has high validity and reliability [24,25,26], being one of the most used linear methods [27]. The SRT was initially described by Wells & Dillon in 1952 [28] and consists of sitting the subject with the legs together and the knees extended, placing the feet in 90° flex against a drawer designed for that purpose. In our study, the measurement drawer used (PO Box 1500, Fabrication Enterprises Inc., White Plains, NY, USA) had a scale attached to the top. The participants were evaluated with sportswear (shirt and shorts) and without shoes, sitting on the floor with the legs together, knees extended, and feet perpendicular to the floor in contact with the measuring box, pressing the heels against it. Then, with palms of both hands down and with the fingers stretched, the participants had to get as far as possible by sliding the hands across the top of the ruler, keeping the position for at least two seconds. The sit and reach test score in centimeters was registered as the final position of the fingertips on or towards the ruler. Higher scores indicated better performance. The test was performed twice, and the best score was retained as described elsewhere [29,30,31,32].

In addition, we performed the deep flexion of the trunk (DF) test following the protocol described by Zurita et al. [14] and used in previous studies elsewhere [15,33] to evaluate the flexor capacity, determined by the modifications in the rachis during the movement of deep anterior flexion of the trunk. For the DF test, the participants were placed in a standing position with legs separated and barefoot on a wooden platform (0.76 by 0.88 m), matching the heels of both feet with the line indicating the value 0 of the scale. In the execution of the test of deep flexion of the trunk, knees are flexed, the hands pass between the two legs trying to reach as far back as possible on the ruler millimeter. 

### 2.3. Procedure

In the test measurement session, body composition was measured prior to the execution of the tests, then participants performed a standard warm up consisting of: (i) 5–10 min of aerobic running and (ii) two sets of standardized static stretching exercises of 30 seconds of duration for each one [15,34]. Bouncing was not allowed and the participants were urged to perform the stretch slowly and calmly. The best of both trials was used for statistical analysis. The tests were conducted in a covered sports hall at the same time and under the same environmental conditions, with a temperature between 22 °C and 24 °C.

### 2.4. Data Analysis

Descriptive data are presented as mean ± standard deviation (SD) and tested for normality by a Kolmogorov–Smirnov test. To analyze the linear relationship between the anthropometric variables and the flexibility tests, the Pearson correlation coefficient was used. The multifactorial ANOVA was used to compare the results obtained in the tests (DF and STR) between the different groups (by federated vs non-federated), (by age categories) and (by BMI categories). If there were significant differences between the groups, then a multiple comparison test was performed a posteriori, correcting by the Bonferroni method. All analyzes were performed with the statistical software IBM SPSS v.22 and the level of statistical significance was established at *p* < 0.05.

## 3. Results

### 3.1. Participant Characteristics

In total, 234 male students participated in this study aged between 11 and 18 years (soccer federated students = 107). The characteristics of the included participants are summarized for the whole group and divided by age categories in Table 1, and classified as non-federated students or soccer sport-federated students. The mean (±SD) age of the adolescent students was 13.9 ± 1.8 years for the whole group, 13.4 ± 1.8 years for non-federated students, and 14.4 ± 1.8 years for soccer sport-federated students. BMI significantly differed between age groups (F(3230) = 11.38; *p* < 0.001; η^2^_p_ = 0.13) while federated and non-federated groups had similar BMI values showing some trends; post-hoc comparisons are presented in Table 1. 

### 3.2. Hamstring Flexibility: Non-Federated (G1) vs. Federated Students (G2)

The mean differences for hamstring flexibility (DF and SRT) are presented divided by G1 and G2 in Table 2. The federated group showed higher scores for the DF test (F(1233) = 2.28; *p* = 0.047; η^2^_p_ = 0.11) and SRT test (F(1233) = 9.83; *p* = 0.002; η^2^_p_ = 0.45) than non-federated students for the whole sample (6.2% and 75.8%, respectively).

### 3.3. Hamstring Flexibility: By BMI Categories (UW, NW, OW and OB)

There were significant differences between BMI groups for the scores of the DF test (F(3230) = 14.29; *p* < 0.001; η^2^_p_ = 0.16) but not for the SRT test. Post-hoc comparisons are shown in Table 3. The UW group obtained greater values for DF score for 23.9% and 37.6% compared to OW and OB, respectively (*p* < 0.001). The NW group also obtained greater values for DF score for 20.0% and 34.4% compared to OW and OB, respectively (*p* < 0.001). 

### 3.4. Hamstring Flexibility: By Age Categories

The age categories consisted of four groups: (i) 11–12 yrs, (ii) 13–14 yrs, (iii) 15–16 yrs and (iv) 17–18 yrs. There was no significant main effect of the age groups. Moreover, multifactorial ANOVA showed no significant interactions between any factors.

### 3.5. Correlations

**Figure 1:**
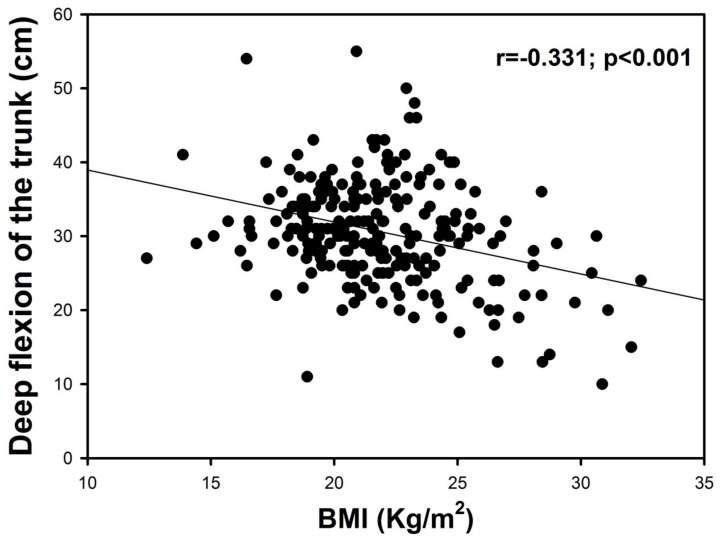
Relationships between body mass index (BMI) and deep flexion on the trunk test.

In the univariate analysis, there is a correlation between both tests of flexibility (DF and SRT) (r = 0.4, *p* < 0.001). BMI was negatively correlated with the DF test (r = −0.3, *p* < 0.001, Figure 1) but not with SRT (*p* = 0.198). These correlations were maintained even when 1 possible confounder (age) was added to the multivariate analysis (r = 0.4, *p* < 0.001 for DF and SRT; r = −0.4, *p* < 0.001 for BMI and DF; r < 0.1, *p* = 0.642 for BMI and SRT adjusted by age). Moreover, there was a positive correlation between SRT score and age (r = 0.2; *p* = 0.011).

## 4. Discussion

The present study reports that the practice of soccer as a federated sport during adolescence increases hamstring flexibility measured trough DF and SRT tests compared to students who only perform physical activity during curricular classes. These differences were more pronounced during the first stages of adolescence, especially in SRT test, but were lost after 15 years old. Moreover, an inverse correlation between weight status measured by BMI and hamstring flexibility level assessed by the DF test has been observed in our data, but not with the SRT test. Accordingly, there is a correlation between BMI and DF score in the whole group.

Despite the fact that one of the aims of the contents of the curriculum in secondary education is the improvement of flexibility, we have found that adolescents who practiced soccer in a federated way had greater scores both in the DF and SRT tests compared to students without practice of any extracurricular sport. These differences in hamstring flexibility might be explained for the inclusion of specific training for flexibility in soccer training sessions, normally in both the warm up and in the final part of the session. Hence, it seems that the time spent on flexibility in schools is too limited to see improvements of this content in the secondary education curriculum. An intervention of 9 weeks with only 3 min of hamstring stretches during physical education (PE) classes found that hamstring flexibility, measured with SRT test, was improved in the experimental group by 6.4% compared to the control group who followed the standard PE class program [35]. Moreover, it has been shown that significant improvements in flexibility occur after a 6-week intervention plan in children and adolescents outside of school [36,37]. In agreement, our adolescents who practice soccer outside the school for at least two years also showed greater flexibility than the control. To our knowledge, this is the first time that the flexibility of adolescent students has been compared with a group of students who practice soccer in a federated way. There is only one study performed with 449 male amateur soccer players, but with adults age with 24.5 ± 3.8 years old. In this study, the score of hamstring flexibility measured with the SRT test was lower compared to other athletes [37,38] or recreationally active young adults [23]. 

It has been shown than soccer training can reduce muscle flexibility in adults due in part to the fact that players had played soccer for several years [18,39,40,41]. Our results show that federated students had higher hamstring flexibility than non-federated students, especially during the first stage of adolescence, but this difference was lower after 15 years old (data not significant). Similarly, increased flexibility performance has been observed with ageing during adolescence, with a plateau from 16 years of age in boys [42]. According to that, we had a positive correlation between SRT score and age in our study.

The present study reported an inverse correlation between weight status and flexibility level assessed by the DF test. However, there was not a significant correlation between weight status and SRT, which concurs with other studies [10,11,12,43]. Some authors have concluded that the SRT test has a moderate validity when it is used only as an assessment of the flexibility of the muscles of the back of the knee, but it seems that it cannot correctly assess the flexibility of the back and in particular of the lower part of the back [33]. Given that flexibility is specific to each joint, and only one measurement cannot indicate the level of flexibility of all joints, additional flexibility tests are necessary to include the flexibility of the back [2]. The fact that the DF test included more of the flexibility of the back could explain the correlation between weight status and DF test and not with SRT test. The anthropometric factors are one of the main variables that could distort the results obtained in the SRT test, the range of movement of the different joint involved, and the disposition of the spine could affect the correlation with BMI [14].

We observed that overweight and obese students had significantly lower scores in the DF test than their underweight and normal weight counterparts. Normal weight and underweight groups had similar performance in both tests. Given the way that this test is performed, the excess of body fat mass in the trunk might limit the range of motion and therefore the flexibility of in this area. Whether the observed statistical differences (~5 cm) across weight status categories have clinical interest remains to be elucidated. However, both federated and non-federated students had similar BMI even when separated by age categories.

It would be appropriate in these ages to propose to physical education teachers and coaches/trainers a specific training plan of flexibility to avoid the fall in the values of flexibility. It would be necessary to establish a good stretching program of the hamstring muscles as a basis to avoid future injuries in soccer [24]. The training and improvement of flexibility at these ages can prevent injuries in young sportsmen, and it is highly recommend to introduce specific training of flexibility in ages between 10 and 15 years. Despite this, Van Doormal et al. [44] showed no relationships between hamstring flexibility and hamstring injuries in male amateur soccer players. 

The strength of this study is that we carried out two types of hamstring flexibility tests, when normally only one is used (SRT). This article suggests that practicing soccer in a federated way can benefit hamstring flexibility and suggests that educational system is not enough, and probably the number of hours of physical education should be increased to improve the physical condition of students. However, the main limitation of this study is that it is a cross-sectional cohort study, with longitudinal or intervention studies being of great interest for future research to observe the benefits on hamstring flexibility. It would also be interesting to compare the effect of different modalities of federated sports on hamstring flexibility.

In conclusion, our study shows that federated soccer players have higher hamstring flexibility measured with two different tests (DF and SRT), which are attenuated from 15 years old. Moreover, when the participants were divided by BMI categories, the underweight and normal weight students had higher scores in the DF test compared to overweight and obese students. Accordingly, BMI was correlated with hamstring flexibility measured with the DF but not with the SRT test. 

## Figures and Tables

**Table 1:** Characteristics of the participants for the whole group (n = 234) and for the created subgroups according to participants’ age, divided into non-federated students (n = 127) and sport-federated students (n = 107).

Variables	Age Categories	ALL	N	Non-Federated	N	Federated	n	Sig
Age (years)	Total	13.9 ± 1.8	234	13.4 ± 1.8	127	14.4 ± 1.8	107	ns
	11–12 yrs	11.7 ± 0.5	67	11.6 ± 0.5	48	11.7 ± 0.3	19	ns
	13–14 yrs	13.5 ± 0.5	79	13.5 ± 0.5	35	13.5 ± 0.5	44	ns
	15–16 yrs	15.4 ± 0.5	70	15.3 ± 0.5	41	15.6 ± 0.5	29	ns
	17–18 yrs	17.5 ± 0.5	18	17.3 ± 0.6	3	17.5 ± 0.5	15	ns
Height (cm)	Total	167.4 ± 10.5	234	165.3 ± 11.4	127	169.9 ± 8.6	107	Ns
	11–12 yrs	156.0 ± 8.7	67	154.7 ± 8.0	48	159.4 ± 9.7	19	**0.023**
	13–14 yrs	170.6 ± 7.5	79	169.5 ± 8.6	35	171.5 ± 6.4	44	Ns
	15–16 yrs	172.7 ± 6.4	70	173.3 ± 6.7	41	171.9 ± 6.0	29	Ns
	17–18 yrs	174.9 ± 6.9	18	176.0 ± 4.6	3	174.7 ± 7.4	15	Ns
Body mass (kg)	Total	61.9 ± 13.0	234	61.0 ± 14.5	127	62.9 ± 11.0	107	Ns
	11–12 yrs	49.9 ± 10.5	67	50.1 ± 11.1	48	49.2 ± 9.1	19	Ns
	13–14 yrs	63.1 ± 9.4	79	64.4 ± 10.9	35	62.0 ± 7.9	44	Ns
	15–16 yrs	69.0 ± 10.4	70	69.6 ± 12.2	41	68.2 ± 7.3	29	Ns
	17–18 yrs	73.2 ± 11.4	18	76.3 ± 21.5	3	72.5 ± 9.5	15	Ns
BMI (kg·m^−2^)	Total	21.9 ± 3.3	234	22.1 ± 3.7	127	21.7 ± 2.9	107	ns
	11–12 yrs	20.4 ± 3.4 *	**67**	20.8 ± 3.6	48	19.3 ± 2.9	19	**0.091**
	13–14 yrs	**21.7 ± 2.9&**	**79**	22.4 ± 3.3	35	21.1 ± 2.5	44	**0.069**
	15–16 yrs	23.1 ± 3.0	70	23.1 ± 3.5	41	23.1 ± 2.2	29	ns
	17–18 yrs	23.9 ± 3.2	18	24.6 ± 6.2	3	23.8 ± 2.6	15	ns

Note: the values are mean ± SD; BMI: body mass index; ns: *p* > 0.10; significant differences and trends in the Bonferroni’s comparisons between non-federated vs. federated are expressed in bold in the last column. Significant differences for BMI are expressed in bold (* *p* < 0.001 respect to 15–16 and 17–18 years and & *p* < 0.05 respect to 15–16 and 17–18 years).

**Table 2:** Bonferroni post-hoc comparisons between federated and non-federated students in flexibility tests.

Test	ALL (n = 234)	Federated (n = 127)	Non-Federated (n = 107)	*p*-Value
DF (cm)	30.6 ± 7.1	29.7 ± 8.0	31.6 ± 5.6	**0.048**
SRT (cm)	6.9 ± 7.6	5.2 ± 8.3	9.1 ± 6.1	**0.001**

Note: the values are mean ± SD; DF: deep flexion of the trunk test; SRT: sit and reach test. Significant differences are expressed in bold.

**Table 3:** Bonferroni post-hoc comparisons between body mass index categories in flexibility tests.

Test	ALL (n = 234)	Underweight (n = 28)	Normal Weight (n = 168)	Overweight (n = 32)	Obesity (n = 6)
DF (cm)	30.6 ± 7.1	33.1 ± 6.2 *^,#^	31.5 ± 6.5 *^,#^	25.2 ± 6.8	20.7 ± 7.3
SRT (cm)	6.9 ± 7.6	3.4 ± 8.1	7.7 ± 7.3	6.7 ± 7.9	4.7 ± 10.1

Note: the values are mean ± SD; DF: deep flexion of the trunk test; SRT: sit and reach test. * *p* < 0.05 respect to obesity, ^#^ *p* < 0.05 respect to overweight.
